# Surfalize: A Python Library for Surface Topography and Roughness Analysis Designed for Periodic Surface Structures

**DOI:** 10.3390/nano14131076

**Published:** 2024-06-24

**Authors:** Frederic Schell, Christoph Zwahr, Andrés F. Lasagni

**Affiliations:** 1 Fraunhofer Institute for Material and Beam Technology IWS, Winterbergstraße 28, 01277 Dresden, Germany; 2 Institut für Fertigungstechnik, Technische Universität Dresden, George-Bähr-Str. 3c, 01069 Dresden, Germany

**Keywords:** surface roughness, periodic microtextures, Python library, confocal microscopy, Surfalize, ISO 25178

## Abstract

Surface roughness measurement is an integral part of the characterization of microtextured surfaces. Multiple established software packages offer the calculation of roughness parameters according to ISO 25178. However, these packages lack a specific set of features, which we hope to address in this work. Firstly, they often lack or have limited capabilities for automated and batch analysis, making it hard to integrate into other applications. Secondly, they are often proprietary and therefore restrict access to some potential users. Lastly, they lack some capabilities when it comes to the analysis of periodic microtextured surfaces. Namely, common parameters such as the peak-to-valley depth, spatial period and homogeneity cannot be calculated automatically. This work aims to address these challenges by introducing a novel Python library, *Surfalize*, which intends to fill in the gaps regarding this functionality. The functionality is described and the algorithms are validated against established software packages or manual measurements.

## 1. Introduction

The characterization of functional surfaces plays a crucial role in relating surface functionality to surface morphology [1,2]. For instance, surface roughness influences mechanical [3], biological [4,5,6,7], optical [8], tribological [9], thermal [10] and wetting [11] properties.

The ISO 25178 international standard [12] establishes the framework for 3D surface texture analysis, encompassing both contact and non-contact measurement methods. One of its key features is the comprehensive set of parameters it defines for quantifying surface roughness. The parameters are categorized into groups, such as the height parameters that are related to the amplitude [13], the functional parameters that are defined based on the areal material ratio function (also known as Abbott–Firestone curve) [14] and the hybrid parameters that are computed from surface envelope and gradients [13]. These parameters are crucial for accurately characterizing surface topography, ensuring consistency and reliability in quality control and performance assessments, and are increasingly adopted across various industries [15].

In the domain of microstructured surfaces, tools such as confocal microscopes are often used to measure the three-dimensional topography of textured parts. However, the characterization of these surfaces often relies on a limited selection of expensive and proprietary software packages, most prominently, the software MountainsMap [16,17,18,19,20]. As such, these packages may not be available to all researchers or may be restricted to a single device license per institution, which can severely impact accessibility and productivity. This creates a demand for a free, open-source software solution. 

The only prominently available open-source alternative software, Gwyddion [21], while quite comprehensive in other regards, does not currently provide the whole parameter set defined by ISO 25178, lacking for instance the hybrid, functional and functional volume parameters derived from the Abbott–Firestone curve. 

Furthermore, the existing software tools lack efficient automation and batch processing capabilities, which are essential for handling the growing demand for large datasets in machine learning applications. Given that Python has become the de facto standard for scientific computing and data analysis [22], a Python-based surface analysis library could significantly benefit researchers by seamlessly integrating into existing scientific Python workflows. Although Python bindings are available for Gwyddion, they are restricted to the no longer officially supported Python 2 (due to Gwyddion being written using GTK+ 2) and require the installation of Gwyddion binaries. 

Lastly, proprietary software packages struggle with accommodating custom functionalities demanded by specific research domains. For instance, in the context of laser-based fabrication of periodic surface microtextures using techniques such as direct laser writing (DLW) or direct laser interference patterning (DLIP), parameters such as the peak-to-valley structure depth [23,24], the spatial period [25,26] and the texture homogeneity [27,28,29] are often referenced in publications and mostly computed by hand. An open-source solution that can be easily extended by anyone to compute custom parameters could therefore be beneficial to many researchers.

To address these limitations, this work introduces *Surfalize*, a Python 3 library designed to streamline surface analysis tasks. By leveraging the accessibility and extensibility of Python, the library aims to offer researchers an intuitive, versatile and customizable toolset for characterizing microstructured surfaces efficiently and comprehensively.

## 2. *Surfalize* Description

*Surfalize* is an open-source Python 3 library for the analysis of 3D confocal microscope/white light interferometer data of microtextured surfaces. Its aim is to facilitate the evaluation of surface topography data regarding the computation of surface roughness parameters according to the ISO 25178 standard [12] as well as other surface properties associated with periodic surface microtextures. The library is designed with two different purposes in mind. Firstly, it integrates well with Jupyter Notebooks [30], which is based on IPython [31], and allows for an interactive programming in cells with the ability to plot any output directly under the cell, similar to the design of Matlab. Thereby, it can be integrated into the workflow that many scientists already use for data analysis, combining Jupyter Notebooks with the scipy stack of Python (numpy, matplotlib, pandas, scipy, scikit-learn). Secondly, *Surfalize* is designed to integrate well into other software toolchains. For instance, AI applications with regard to surface topography could greatly benefit from an extendable open source library for batch computation of surface roughness. Moreover, the library is easily extensible and can be adapted to suit all kinds of computational requirements. It implements various common file formats for topographic microscope measurements and can thereby easily grant access to the underlying data for custom computations. In addition, *Surfalize* offers a variety of operations and characterizations of surface topographies, as well as plotting and batch processing functionality. These are described in the following.

### 2.1. Operations

Surface objects implement a set of operations that are computed on the topography data. Those operations include:

- Interpolation of non-measured points;
- Plane leveling;
- Spatial filtering;
- Rotation;
- Texture alignment;
- Cropping;
- Profile extraction;
- Outlier removal;
- Thresholding based on areal material ratio curve.

### 2.2. Quantitative Characterization

For the quantitative characterization, ISO 25178 [12] defines a comprehensive set of parameters to quantify and describe the three-dimensional surface texture of materials. These parameters are grouped into families that capture different aspects of surface characteristics: height parameters describe vertical deviations, spatial parameters focus on the distribution and arrangement of features, hybrid parameters combine height and spatial information, and functional parameters assess the surface's ability to perform specific functions, such as load-bearing or fluid retention [2]. Additionally, feature and surface texture parameters provide detailed insights into specific surface features and their statistical properties. The proposed library implements the most common surface roughness parameters defined in the standard, as well as additional quantities in the context of periodic microtextures:

- Height parameters: Sa, Sq, Sz, Sv, Sp, Sku, Ssk;
- Hybrid parameters: Sdq, Sdr;
- Functional parameters: Sk, Spk, Svk, Smr1, Smr2, Sxp, Smr(c), Smc(mr);
- Functional volume parameters: Vmp, Vmc, Vvc, Vvv;
- Spatial parameters: Sal, Str;
- Period texture parameters: spatial period, structure depth, aspect ratio, orientation, homogeneity.

### 2.3. Plotting

Surface objects implement a set of plotting capabilities. These can be used to inspect the surface visually in the spatial domain and in the frequency domain, including:

- Plotting surface topography in 2d;
- Plotting the Fourier transform;
- Plotting the autocorrelation function;
- Plotting of the Abbott–Firestone curve;
- Plotting of the visual parameter study of the functional parameters.

### 2.4. Batch Processing

*Surfalize* is equipped with a module for batch processing that enables the application of surface operations and parameter calculations to a large set of topography files at once. It leverages multi-core processing to apply the processing in parallel where possible, and it returns the extracted values in a tabular format that can be saved to disk as an Excel spreadsheet or CSV (or similar formats).

### 2.5. File Formats

The developed Phyton library currently supports file formats from a number of confocal microscope and white light interferometer manufacturers. These file formats are listed in Table 1 and usually encode a two-dimensional array of height data that lies on a regular grid. A surface represented in *Surfalize* is only characterized by its height data as a 2D array as well as the lateral and vertical pixel resolution. Optionally, image layers, such as grayscale, RGB or intensity layers, as well as metadata can be read from the file. Moreover, *Surfalize* supports exporting to file formats which are designed to hold device and manufacturer independent data, such as the SURF and SDF file standards. Other file formats, which are device specific in nature, such as the VK family, are only supported for file reading. Lastly, extensions for other file formats can easily be added due to the modular design of the file readers.

## 3. Implementation

In this section, the implementation of the algorithms and roughness parameter calculations is discussed, including typical surface parameters (from ISO 25178) as well as period, depth, orientation and homogeneity of periodic structures.

### 3.1. ISO 25178 Surface Roughness Parameters

The height parameters are implemented using the discrete approach without interpolation in the same manner used by most commercially available software. In order to speed up the calculation, all height parameters are implemented in Cython [32] and calculated in a single loop over the array containing the surface data, thereby achieving a hundredfold increase in processing speed compared to the pure Python implementation.

For the purpose of determining Sdr, the surface area of the topography is calculated according to the approach outlined in [33], which is officially referenced in the ISO standard 25178. This algorithm spans two triangles ABC and ADC between each set of four neighboring points A, B, C, D. The total surface area is then approximated as the sum of all triangles. 

**Figure 1:**
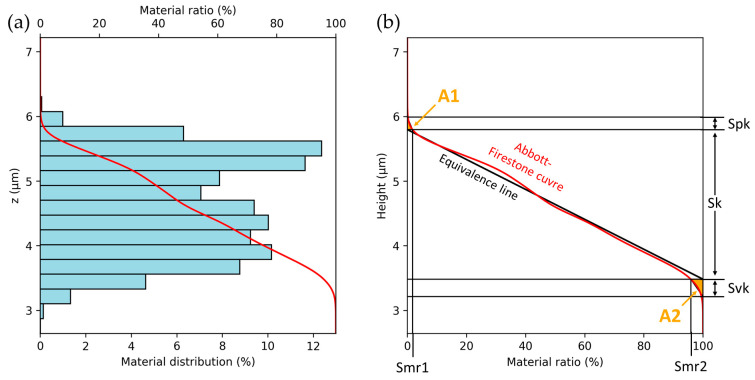
Abbott–Firestone curve (**a**) of an exemplary micro-textured surface and (**b**) visual study of the functional parameters on the Abbott–Firestone curve, including the core roughness depth Sk, the reduced peak height Spk, the reduced dale height Svk and the material ratios Smr1 and Smr2.

The functional and functional volume parameters are derived from the discrete Abbott–Firestone curve, as plotted in Figure 1a. The Abbott–Firestone curve, also called the areal material ratio curve, represents the cumulative surface area above a height c as the function of the height. It is calculated by binning the height values of the surface topography and calculating the cumulative height distribution. Some discretization error occurs due to the finite bin width. The functional parameters are then derived in accordance with ISO 25178 [12] from the curve by constructing a straight line of equivalence as a secant line through two points of the curve spaced apart by a material ratio of 40%, so that a minimal absolute slope is reached, as shown in Figure 1b. If more than one pair of points exhibits the same slope, the first occurrence starting from a material ratio of 0% is utilized. The core height Sk is calculated as the height difference between the value of the equivalence line at both 0% and 100% material ratio. The material ratios Smr1 and Smr2 are evaluated at the intersection of the Abbott–Firestone curve with the horizontal lines at the start and end height of the equivalence line. The reduced dale and peak heights (Svk and Spk) are calculated by constructing triangles with the same areas as the area enclosed by the Abbott–Firestone curve and the vertical axis at 0% and 100% material ratio, respectively. These triangles with the areas A1 and A2 (see Figure 1b) are defined to have a horizontal side length corresponding to Smr1 and (100%—Smr2), respectively. Their vertical side length can therefore be determined from the aforementioned values and is equivalent to Spk and Svk, respectively. This approach makes the calculation of these parameters more robust against large outliers.

**Figure 2:**
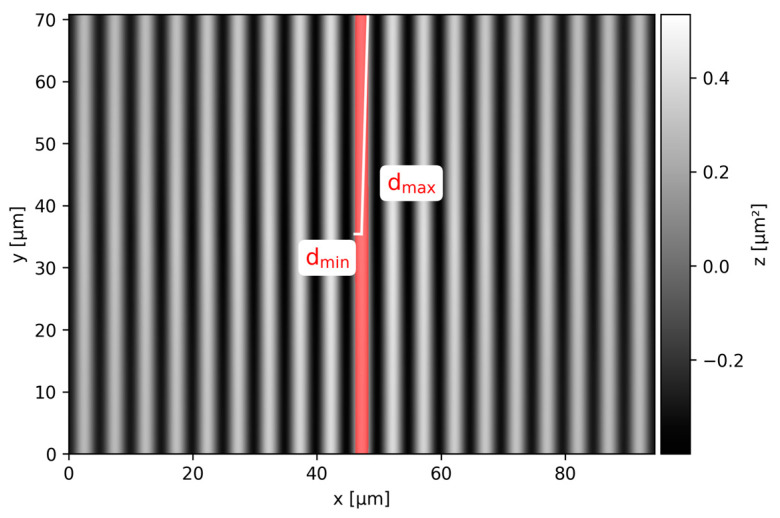
Normalized autocorrelation function of a periodic textured surface computed from the discrete Fourier transform. The area marked in red identifies the region where the values of the autocorrelation lie above the threshold values of 0.2. The distances of shortest decay (d_min_) and longest decay (d_max_) to the threshold value are indicated in white.

The spatial parameters Sal and Str are calculated from the autocorrelation function (ACF, shown in Figure 2), which is computed by means of fast Fourier transform (FFT). The contiguous region of the ACF around its center, where the values lie above a specific threshold value s, is determined (indicated with red color). The parameter s indicates a fraction of the maximum ACF value in the center and shall assume a value of 0.2 by default, according to the standard. The distance of shortest decay of the ACF to the threshold value (d_min_) is equivalent to the parameter Sal. The parameter Str is then computed as the ratio of the distance of shortest decay to the distance of longest decay (d_max_).

### 3.2. Surface Texture Period

**Figure 3:**
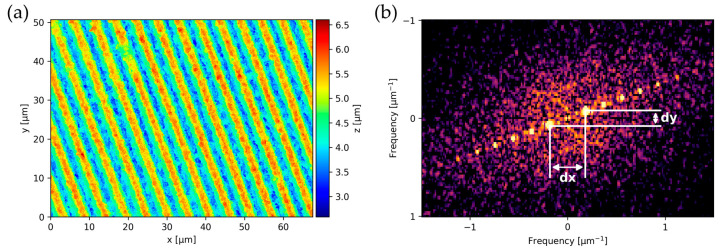
(**a**) Representative DLIP-produced periodic surface structure (with a period of 5 µm) and its calculated (**b**) center of the discrete Fourier transform with a 20° orientation towards the vertical axis.

The texture period (Λ) is retrieved by calculating the 2D discrete Fourier transform (DFT), using the FFT algorithm (see Figure 3). In order to eliminate the zero-peak, the data is centered around the mean by subtracting the mean data height for the array. Then, both x- and y-frequencies of the DFT peaks are computed by a peak-finding algorithm. The peaks are sorted by prominence in descending order and the first two peaks are extracted, corresponding to the dominant texture spatial frequency. The hypotenuse Λ of the peak distance in x and y is calculated and the period is obtained as the inverse of half the peak distance as follows:

(1) $$\Lambda =\frac{2}{\sqrt{dx^{2}+dy^{2}}}$$

### 3.3. Texture Depth

**Figure 4:**
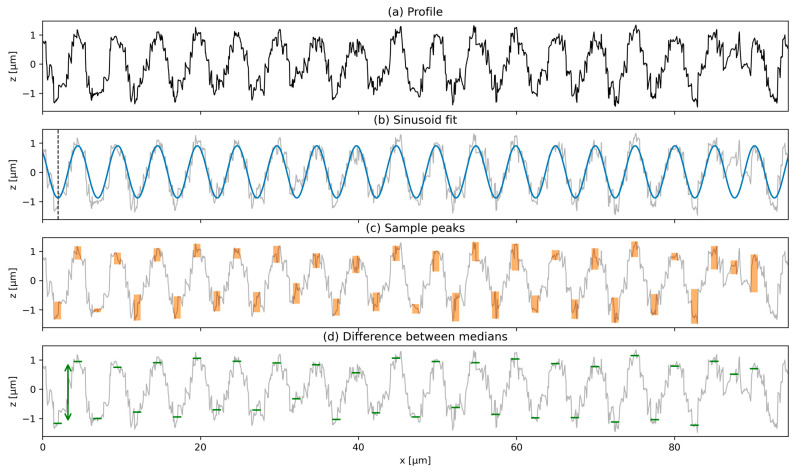
Depiction of the algorithm for peak-to-valley depth estimation shows: (**a**) an exemplary profile, (**b**) a sinusoid that is fitted to the profile data, (**c**) the evaluation area around the peaks, and (**d**) the median of the profile data inside the evaluation area.

Several publications that focus on line-like periodic surface textures evaluate the peak-to-valley structure depth and rely on manual tools for this purpose. The algorithm presented here aims to approximate a reliable peak-to-valley depth value of periodic surface textures with sinusoid properties fully automatically. This is accomplished by fitting a sinusoid (see Equation (2)) to the profile, using a least-squares optimization, as shown in Figure 4a.

(2) $$f\left(x\right)=a\sin\left(\frac{x-x_{0}}{p}2\pi \right)+y_{0}$$

To improve fit convergence to the global minimum, an initial guess of parameters is provided, where the amplitude *a* is set to the absolute distance between the maximum and minimum profile values, the period *p* is estimated from the DFT, and the vertical offset $y_{0}$ is set to the average profile value. The position of the first extremum $x_{fe}$ (first peak of the sinusoid inside the definition area for x > 0, indicated by the dashed vertical line in Figure 4b) of the sinusoid is computed using Equation (3), where the open square brackets indicate the floor function:

(3) $$x_{fe}=\left(x_{0}+\frac{p}{4}\right)-\frac{p}{2}\left\lfloor \frac{x_{0}+\frac{p}{4}}{\frac{p}{2}}\right\rfloor$$

The extrema (peaks and valleys) positions of the sinusoid can then be calculated over the whole definition area of the profile. The profile is then sampled around the extrema of the sinusoid with a predefined lateral extent of 0.2⋅p, indicated by the orange rectangular areas marked in Figure 4c. In order to obtain an outlier resistant estimate of the profile value in the sampling area, the median profile value inside the sampling area is calculated, as indicated by the green lines in Figure 4d. This allows the computation of the depth from adjacent pairs of peak and valley median values. By sampling a specified number of equally spaced profiles from the surface, the mean depth and its standard deviation are obtained from the set of all sampled peak-to-valley depths over all sampled profiles.

The advantages of this algorithm over manual measurements consist not only of the significantly larger number of sampled peak-to-valley intervals compared to manual evaluation, but also of taking into account inhomogeneities that would likely be overlooked in manual computation. For example, Figure 4c shows that the algorithm detects the position of a structure peak that should have been a valley and evaluates it, nonetheless.

### 3.4. Orientation of the Periodic Structure

A first estimate of the texture orientation is extracted from the DFT by calculating the angle of the first order peaks towards the horizontal axis. Figure 2 shows the natural logarithm of the DFT windowed by a Hanning window to suppress spectral leakage. An estimate for the angle of the texture can be computed using Equation (4) (which is derived from the values obtained using the analysis described in Section 3.2).

(4) $$\theta_{DFT}=\tan^{-1}\left(\frac{dy}{dx}\right)$$

**Figure 5:**
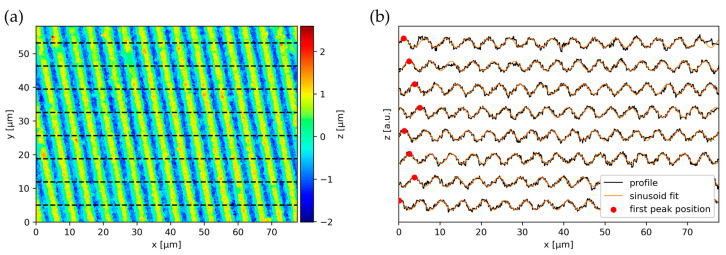
Obtaining the refined orientation from sampled profiles (**a**). The position of the first peaks is indicated by the red marker (**b**). The calculated orientation angle for this pattern was 9.86°.

However, the resolution of the Fourier transform for low frequencies is quite poor, which can result in significant errors on the estimated texture angle. Hence, an additional refinement step is implemented using a similar principle as the texture depth estimation (see Section 3.2). The approach depicted in Figure 5 samples multiple profiles along the texture and fits a sinusoid to them. The sampling is either performed in horizontal or vertical direction, depending on which direction exhibits the larger angle towards the initial estimate of the texture direction obtained from the DFT. For each sinusoid, the position of the first extremum within the definition area is calculated. From these, the distance between two first extrema of adjacent profiles can be calculated. Using a thresholding based on the median distance, the outlier values that correspond to the profiles where the first extremum jumps back by one period are removed. From the cleaned data, the average slope and thereby the angle of the texture can be calculated. For instance, the orientation angle of the periodic structure shown in Figure 5 was 10°.

### 3.5. Texture Homogeneity

**Figure 6:**
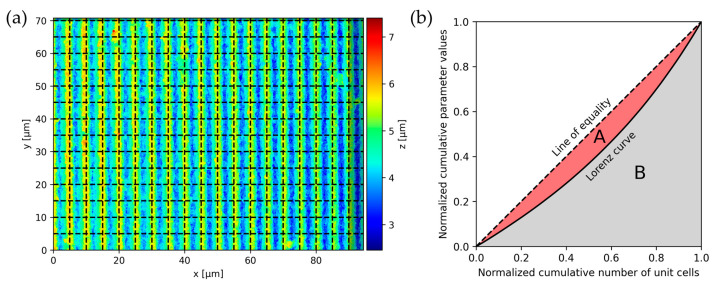
Calculation of homogeneity with (**a**) division of surface into unit cells with the length of the spatial period and (**b**) calculation of the Gini coefficient for each attribute from the Lorenz curve.

The texture homogeneity for periodic structures is assessed based on an implementation of the Gini coefficient algorithm proposed by Lechthaler et al. [27] and also applied by Soldera et al. [28] (the reader is referred to these for a detailed description). The algorithm computes the texture period from the DFT and dissects the surface into unit cells with a side length corresponding to the texture period, as shown in Figure 6a. The parts of the surface that do not form full unit cells (indicated with a dark shade) are ignored.

For each cell, a set of roughness parameters is calculated (by default: Sa, Sdr, Sku), with the restriction that parameters which can assume negative values are excluded due to issues with normalization [34]. From the obtained values, the Lorenz curve is calculated (see Figure 6b), and the Gini coefficient (G) of that parameter is obtained as the ratio of the area enclosed by the line of equality and the Lorenz curve (A), and the area under the line of equality (0.5 – B). The homogeneity H of each attribute is calculated as H = G − 1, and the overall homogeneity is obtained from the mean of the homogeneity of each individual attribute. In this way, a perfect periodic structure has an H value of 1.

## 4. Validation

### 4.1. Validation of Roughness Parameter Calculations

**Figure 7:**
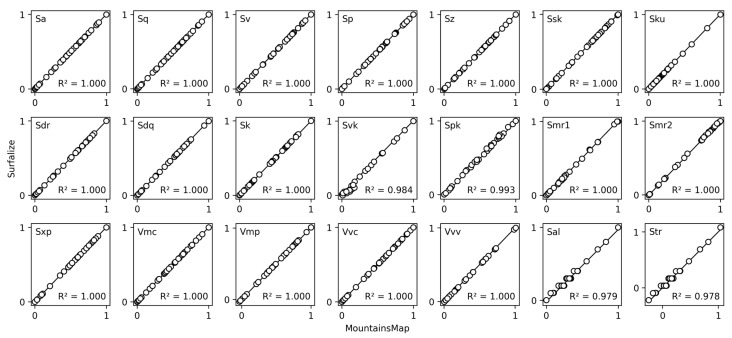
Correlation plots of all surface roughness parameters as calculated by *Surfalize* (y-axis) as a function of the results obtained from MountainsMap (x-axis) for the dataset of 36 periodically textured surfaces presented in [35]. The parameter values are normalized. For each parameter, a coefficient of correlation to the line of equivalence is calculated.

The calculations of the surface roughness parameters according to ISO 25178-2 and ISO 25178-3 were validated against the values obtained from MountainsMap. For this purpose, a dataset presented in a previous work [35] was used, which consists of 36 different line-like periodic textures produced on stainless steel using a laser-based method called Direct Laser Interference Patterning (DLIP). The textures were evaluated by *Surfalize* batch analysis routines. The obtained data for all roughness parameters was normalized and graphed as a function of the corresponding data obtained from MountainsMap as shown in Figure 7. 

The black diagonal line represents the ideal correspondence of roughness values between software packages. To quantify how well the results from both software packages agree, the correlation coefficient (R²) is calculated from the residuals of the obtained data to the ideal line of equivalence (slope of 1, intercept of 0). All height parameters (Sa, Sq, Sv, Sp, Sz, Ssk, Sku) and hybrid parameters (Sdr, Sdq) show perfect agreement between the different software packages with a correlation coefficient of 1.000. The functional parameters (Sk, Svk, Spk, Smr1, Smr2, Sxp) and the functional volume parameters (Vmp, Vmc, Vvv, Vvc) exhibit slight deviations from the reference values, with the lowest coefficient of correlation of 0.984 obtained for Svk. This is related to the comparatively complex calculations compared to the height parameter family. All functional parameters are based on the area material ratio curve, which is computed by binning of the height data. This can lead to discretization errors in the derived parameters, that depend on the chosen number of bins. Furthermore, the central part of the area material ratio curve is approximated with the equivalence line, as described in Section 3.1. Differences in the algorithm for the determination of the equivalence can lead to small differences in its slope, which can lead to noticeable differences in the obtained functional parameters since they are very sensible to the slope and intercept of the equivalence line. However, unsurprisingly, similar differences in calculated roughness values are also observed between established software packages [36]. 

Moreover, it is evident that the parameters Sal and Str are calculated with some form of interpolation in MountainsMap, whereas *Surfalize* does not currently interpolate between the pixels of the autocorrelation function. This explains the observed discontinuous increase of the values for Sal and Str.

### 4.2. Validation of Structure Period and Peak-to-Valley Depth Estimation

**Figure 8:**
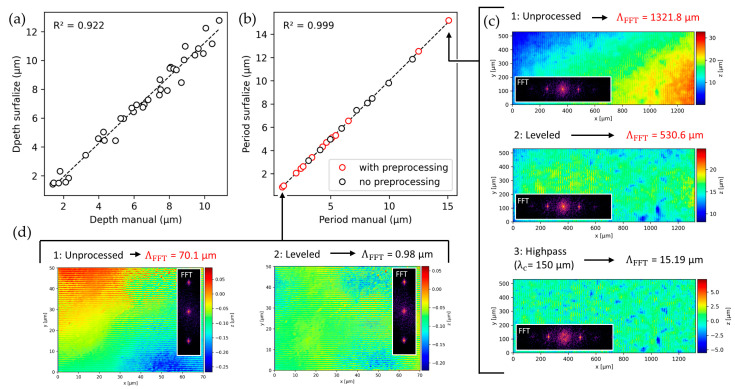
(**a**) Peak-to-valley structure depth of 36 textures calculated by *Surfalize* compared to manually determined. (**b**) Structure period for 23 different textures obtained by *Surfalize* versus manually measured from profiles. The surfaces are leveled and spatially filtered with a highpass filter at a cutoff wavelength of 20 µm, and outliers are removed before determining the period with *Surfalize*. For the red datapoints, the correct value is only computed when these preprocessing steps are applied. (**c**,**d**) Examples of textures with periods of 14.08 µm and 0.96 µm, respectively.

For the peak-to-valley depth, the same dataset used for the roughness parameters was employed. As a reference, the structure depth was measured manually as the mean value over at least 10 periods on three different profiles sampled perpendicular to the grooves. For the validation of the spatial period, 23 different groove textured samples with periods ranging from 0.85 to 15.19 µm were used. The period was also measured manually as a reference over 10 peaks. The depth algorithm performs well compared to the manual measurements, with a coefficient of determination of 0.92 indicating good correlation of the values, as shown in Figure 8a. The remaining variance in the values can be explained by the lower sampling rate of the manual method compared to *Surfalize*, which samples significantly more peaks and is not prone to sampling biases, which may result in picking only homogeneous parts of the texture with pronounced grooves instead of areas which feature defects. It can therefore be assumed that the deviations of the *Surfalize* values are likely due to issues in the manual determination and the calculated values are in fact a more accurate representation of the actual depth.

The determination of the texture period, shown in Figure 8b, reveals that for the investigated textures, *Surfalize* can reliably determine the spatial period based on the Fourier transform. However, the datapoints marked in red required preprocessing in order to correctly determine the spatial period. This relates to the presence of low spatial frequency components on the surface that produce larger peaks in the Fourier transform than the actual frequency of the periodic pattern. Such low frequency components could be a linear inclination of the surface or a waviness component that could be related to a machining process used to achieve the surface finish of the material. To eliminate potential inclination, a leveling operation should be performed. To eliminate waviness, a high-pass filter should be applied. For the results shown in Figure 8b, the preprocessing included plane-leveling, high-pass filtering with a cutoff of 20 µm, as well as the removal of outliers.

This process is exemplified in Figure 8c for a texture with a period of 15.08 µm and in Figure 8d for a texture with 0.96 µm. In both cases, the presence of a linear inclination as well as a low frequency waviness component result in significant overestimations of the spatial period due to the highest peak of the DFT being located in the center. After leveling and application of an appropriate high-pass filter, the algorithm correctly detects the peak corresponding to the texture period. In general, users should be aware of the presence of waviness and inclinations in their data and select appropriate preprocessing. 

### 4.3. Alignment

**Figure 9:**
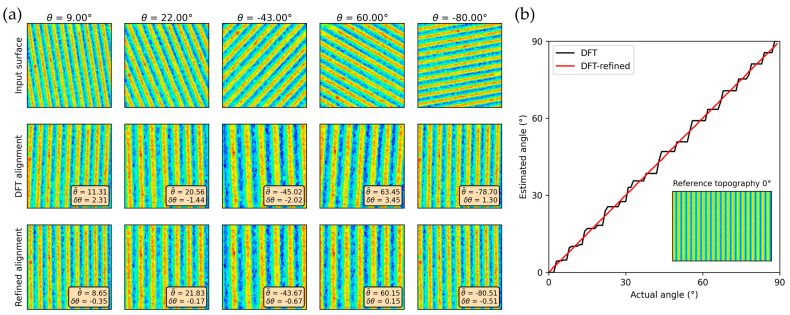
Effects of the alignment algorithm for a reference texture rotated by different angles for (**a**) 5 rotated real textures and (**b**) graph of estimated versus actual angle of a simulated topography. Both the alignment using the purely DFT-based algorithm and the alignment using the refined algorithm are presented. The refined algorithm results in better alignment in all test cases.

The alignment algorithm was tested on a reference texture rotated by different angles. The results are presented in Figure 9a for both the purely DFT-based algorithm and the refined algorithm based on a rotated real texture. The DFT-based algorithm succeeds in aligning the texture roughly with the vertical axis, but exhibits errors between 1.30° for an input angle of −80.0° and 3.45° for an input angle of 60°. Differently, the refined algorithm, although less computationally efficient, results in better alignment across all input angles, with an average error of 0.37° over the five test inputs compared to an average error of 2.1° for the DFT-based alignment. Figure 9b shows the estimated angle of an artificially generated texture with angles from 0° to 90°. It is evident that the refined algorithm does not exhibit large deviations from the equivalence line, whereas the DFT-based algorithm exhibits a stepwise increase of the estimated angle. The average error for the DFT-based algorithm amounts to 1.32°, while the average error for the refined algorithm is only 0.06°.

### 4.4. Homogeneity Validation

The texture homogeneity cannot be validated against established software, since the algorithm for its computation was only recently proposed by Lechthaler et al. [27]. However, due to the known fact that a Gini coefficient of zero represents a distribution of perfect equality of the attributes, a perfectly homogeneous surface must result in a homogeneity factor of one. Increasing introduction of inequality in the distribution of the attributes should yield Gini coefficients closer to one and consequently homogeneity factors tending towards zero. In order to validate the implementation of *Surfalize* against this expected behavior, a perfectly homogeneous line-like surface topography is simulated using Equation (5) with n = 0. Through multiplication of a cosine with the x and y coordinates to the power of n, the distribution of the heights in the array is skewed progressively towards a corner of the topography, thereby increasing the inequality of the distribution.

(5) $$z\left(x,y,n\right)=\cos\left(\frac{2\pi }{\Lambda }x\right)x^{n}y^{n}$$

**Figure 10:**
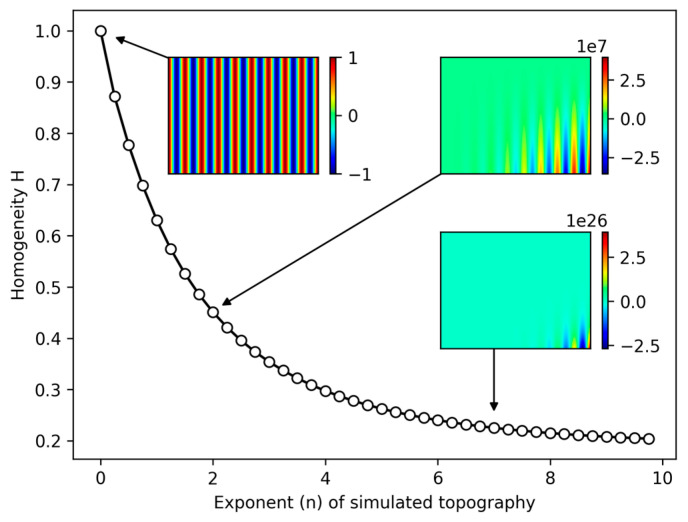
Homogeneity H calculated for different simulated line-like topographies. The horizontal axis represents the exponent n used to calculate the simulated topography with increasingly skewed height distributions for larger powers of n.

Figure 10 shows the calculated homogeneity values for variants of the simulated topography with a period Λ of 10 µm for increasing values of n. The perfectly homogeneous surface for n = 0 correctly results in a homogeneity value of 1.0. For increasing n, the homogeneity decreases continuously and asymptotically approaches a value of 0.2. The observation that the homogeneity does not seem to approach zero can be explained by the utilization of the trapezoidal rule for integrating the area under the Lorenz curve. In a scenario of perfect inequality, only one cell holds the entire cumulated value of the attribute, which should theoretically result in an area of zero in the continuous case. With a finite number of unit cells for which the attributes are evaluated, and with the application of the trapezoidal rule, the integration nonetheless yields some value above zero. Since, in practice, homogeneity values of <0.5 were never observed with any topography tested during development, this effect is negligible. 

## 5. Application Example

A possible application is a quick examination of a parameter space of measured values, using the batch capabilities. Here, an array of five by four line-like DLIP textures with a spatial period of 10.5 µm were produced on Ti-6Al-4V samples, using a two-beam DLIP configuration with a nanosecond-pulsed infrared laser (the same setup is described in detail in [35]). On the vertical axis, the laser fluence is varied between 1.21 J/cm² and 2.67 J/cm², while the number of pulses is increased from five to seventeen pulses per spot with each column on the horizontal axis. 

**Figure 11:**
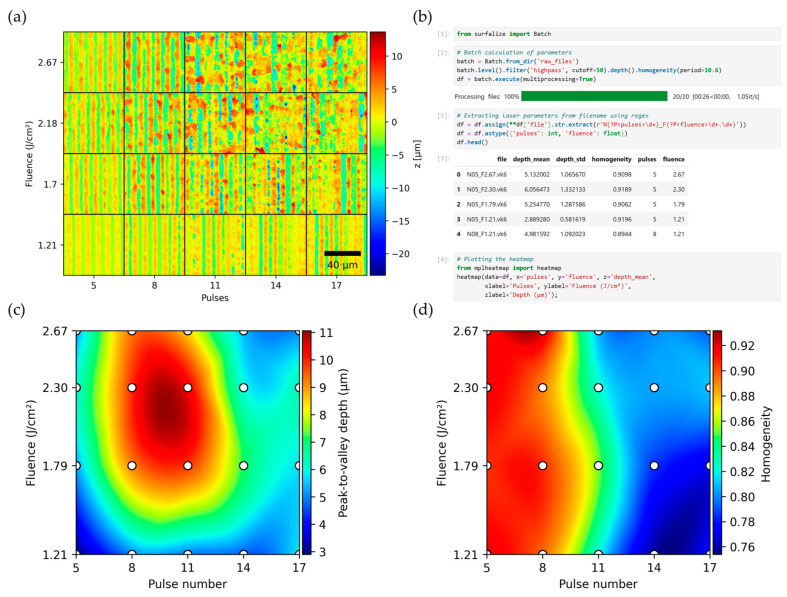
Analysis of an array of surface textures shown in (**a**), fabricated with varying laser fluence and number of pulses per spot. The code that is used to calculate and plot the data is shown in (**b**). The corresponding interpolated heatmaps of the peak-to-valley structure depth (**c**) and the homogeneity (**d**) of the textures computed by *Surfalize* allow for a quick overview of the texture characteristics and enable an easy selection of adequate parameter combinations based on the desired characteristics.

Figure 11a shows a segment of the topography of each measured texture. Using the batch capabilities of *Surfalize* (see code in Figure 11b), the roughness parameters and periodic texture parameters are computed, with only a couple of lines of code being necessary. Before calculation of the parameters, the topographies are plane-leveled and filtered using a Gaussian lowpass filter at a cutoff frequency of 0.5 µm. From the resulting data, the influence of the varied processing parameters can be easily plotted as an interpolated heatmap, revealing the trends of the relevant topographic parameters immediately. In this example, for instance, the parameters should be optimized to achieve a large peak-to-valley depth while maintaining high homogeneity of the textured surfaces. Since it is known that for increasing fluence and number of pulses, the peak-to-valley depth starts to increase up to a maximum but reduces again due to melting effects, which goes along with decreasing texture homogeneity, the optimal values can easily be spotted using the heatmaps shown in Figure 11b,c. In this case, eight pulses and a fluence range of 1.79 to 2.30 J/cm² yield the best results in terms of structure depth, while maintaining homogeneity factors above 0.9, which indicates excellent uniformity.

## 6. Conclusions

This work has outlined the capabilities of the novel Python library *Surfalize* for the characterization of microtextured and rough surfaces. The unique algorithms for the calculation of parameters for laser-textured periodic surfaces were presented. All calculated parameters were validated against established software or manual measurements, using a dataset of laser-textured surfaces. In the future, we plan to support more file formats as well as extend the range of surface roughness parameters and operations. Specifically, feature and fractal parameters, profile roughness and additional filtering techniques are part of the roadmap. In the spirit of open-source development, we welcome any contribution to the Github repository to build a library that works for all researchers in the field.

## Figures and Tables

**Table 1:** File formats currently supported by *Surfalize*.

Manufacturer	Formats	Loading	Saving
Keyence	.vk4	Yes	No
Keyence	.vk6	Yes	No
Keyence	.vk7	Yes	No
Sensofar	.plu	Yes	No
Sensofar	.plux	Yes	No
Digital Surf	.sur (uncompressed) .sur (compressed)	Yes	Yes
Digital Surf	.sdf (ascii) .sdf (binary)	Yes	Yes
KLA	.zmg	Yes	No
Wyko	.opd	Yes	No
Nanofocus	.nms	Yes	No
General	.xyz	Yes	No
Alicona	.al3d	Yes	Yes
Gwyddion	.gwy	Yes	No

## Data Availability

The source code is freely available on Github under https://github.com/fredericjs/surfalize. The data used for the exemplary computations in this manuscript are available upon reasonable request to the authors.
